# Necrotizing fasciitis of the breast after bilateral breast reduction

**DOI:** 10.1093/jscr/rjad230

**Published:** 2023-05-03

**Authors:** David Borg, Kurt Lee Chircop, Duncan Aquilina

**Affiliations:** Department of Plastics and Burns, Mater Dei Hospital, Swatar B'Kara, B'Kara, Malta; Department of Plastics and Burns, Mater Dei Hospital, Swatar B'Kara, B'Kara, Malta; Department of Plastics and Burns, Mater Dei Hospital, Swatar B'Kara, B'Kara, Malta

## Abstract

Necrotizing fasciitis is a rare infection that rapidly progresses through fascial planes. Due to the latter, diagnosis in a timely manner is imperative to ultimately decrease morbidity and mortality. Such a disease process can occur anywhere in the body; however, necrotizing fasciitis of the breast is extremely rare and not well documented in the available literature.

This is a case report about a 49-year-old woman who developed severe necrotizing fasciitis of both breasts following elective bilateral breast reduction. The patient developed a severe soft tissue infection leading to destruction of local tissue and required management in a surgical high dependency unit. This case report outlines the immediate management and the ensuing steps in reconstruction.

Necrotizing fasciitis of the breast is a rare complication of breast reduction surgery. Early recognition and aggressive treatment with broad-spectrum antibiotics, hyperbaric therapy and repeated debridement are essential for successful management. The use of Integra Bilayer Wound Matrix and skin grafting can result in satisfactory outcomes. It is important to obtain tissue samples for culture and sensitivity testing to identify the offending organism in patients with suspected necrotizing fasciitis. This case report highlights the importance of early diagnosis and management of necrotizing fasciitis to prevent morbidity and mortality.

## INTRODUCTION

Necrotizing fasciitis is a rare but potentially life-threatening infection that involves the subcutaneous tissue, fascia and other connective tissues. It is commonly caused by Group A streptococcus, but other bacterial species may also be responsible. The infection can develop in various parts of the body, including the breast, but it is extremely rare. Necrotizing fasciitis of the breast has been reported in very few cases in the literature. Bilateral breast reduction is a common surgical procedure that aims to reduce breast size, relieve symptoms of macromastia and improve breast aesthetics. It is generally considered a safe procedure, with a low risk of complications. However, necrotizing fasciitis is a rare but serious complication that can occur after breast reduction surgery. Despite the low incidence, the severity of the infection and its potential to cause permanent disfigurement and even death make it essential to raise awareness of this condition among healthcare professionals. The objective of this case report is to present a rare case of necrotizing fasciitis of the breast following bilateral breast reduction surgery.

## CASE REPORT

**Figure 1:**
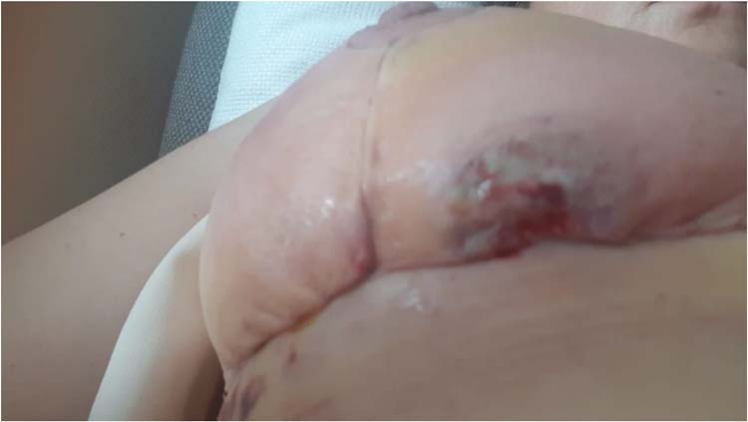
Figure representing the patient’s right breast on presentation

**Figure 2:**
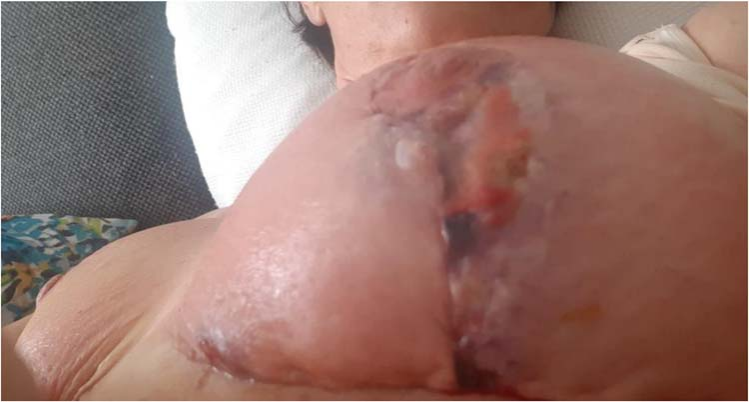
Figure representing superficial skin changes including erythema and skin necrosis of the Left breast on presentation

A 49-year-old previously healthy woman presented to the local Plastics and Burns dressing clinic with fever, bilateral breast tenderness, chills, rigors and increased breast swelling, which started 10 days after undergoing a bilateral breast reduction. The patient underwent an inferior pedicle-wise pattern breast reduction procedure—prior to which she was prescribed 1 g co-amoxiclav twice daily for 7 days. She was reviewed and urgently admitted due to a suspected infected hematoma. The patient was started on ciprofloxacin 400 mg twice daily and clindamycin 600 mg twice daily. The following day, the patient’s condition continued to worsen, with the dressings soiled with pus and patchy areas of skin necrosis with bullae visible on both breasts, as shown in Figs 1 and 2. The patient was urgently planned for theatre for debridement of both breasts. During debridement, it was noted that only the skin was affected, and that the breast tissue and the nipple were all viable. A wound swab was sent for culture and sensitivity.

Postoperatively, the patient was admitted to a high dependency unit, and aggressive treatment with broad spectrum antibiotics was given in accordance with advice from the Microbiology team.

**Figure 3:**
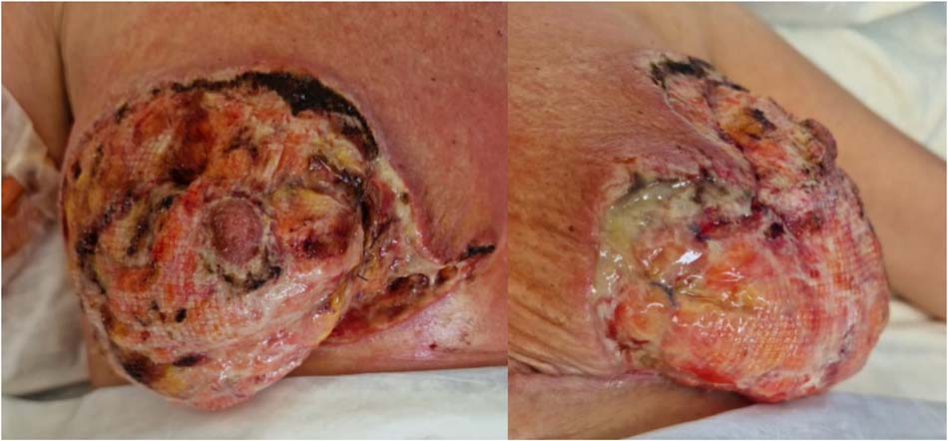
Right and left breasts before second debridement

Three days after the first debridement, the patient started to develop signs of sepsis, namely low urine output, repeated febrile spikes and leukocytosis. Wound inspection showed further spreading of skin necrosis, as shown in Fig. 3. She was started on vasopressors to maintain her mean arterial pressure above 70 mmHg. She was taken to the theatre for the second time for further debridement of necrotic tissue and washout. This time, tissue samples were sent for culture and sensitivity.

**Figure 4:**
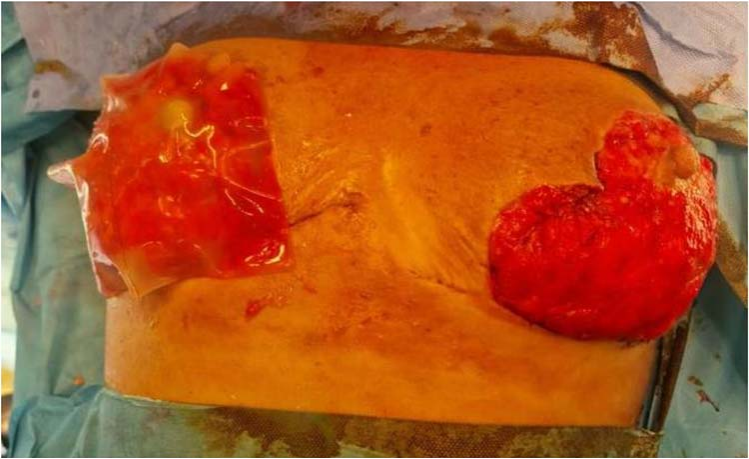
Figure representing bilateral breasts post debridement—this was taken after the third debridement attempt. Application of Integra can be noted on the right breast

Hyperbaric therapy was started on Day 6 of admission, and on Day 15, the patient underwent a third debridement and washout. Skin flaps were advanced as much as possible to cover the areas excised and limit exposure of the underlying soft tissue. On Day 22, the wounds were deemed to be clean enough and both breasts were covered with Integra Bilayer Wound Matrix as the first stage of reconstruction (Fig. 4).

**Figure 5:**
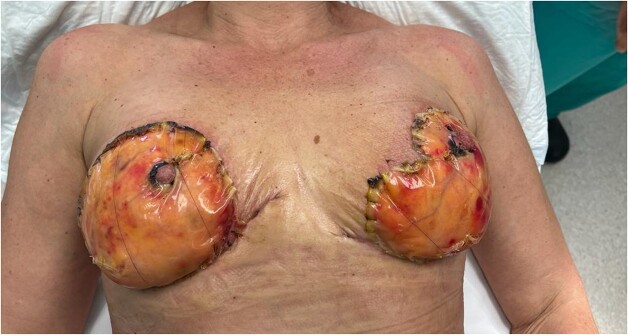
Figure representing clear dermal regeneration under the outer silicon sheath of the Bilayer Matrix at Day 14

She was discharged to the community on Day 29. The first change of dressing was carried out on Day 5 and every 3 days thereafter. No pus was ever noticed during each dressing change, and gradual dermal generation was noted under the outer silicon sheath of the Bilayer Matrix as shown in Fig. 5.

**Figure 6:**
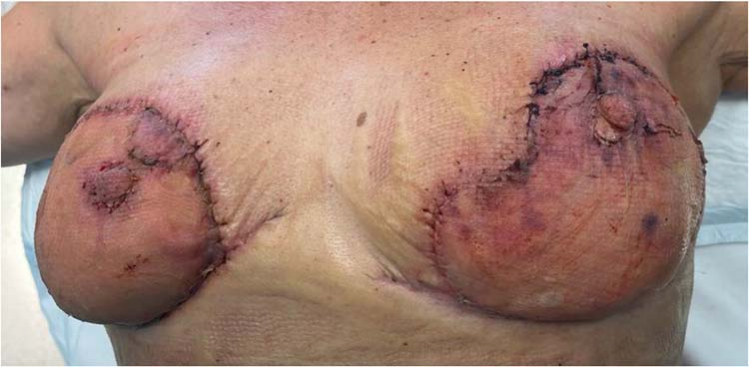
Figure representing 100% graft uptake

After 21 days, the patient was re admitted and underwent removal of the silicon layer, and skin grafting was carried out over the newly formed dermis. The grafts were harvested from the posterior thigh and were not meshed to improve aesthetic appearance. The patient was reviewed regularly following grafting, and there was 100% graft uptake (Fig. 6).

Despite multiple attempts at culturing the offending microorganism, none of the samples submitted to microbiology yielded any results. This was probably because the patient had been on three different types of antibiotics prior to the first debridement. Ideally, a tissue sample should have been sent at the first debridement rather than a wound swab.

**Figure 7:**
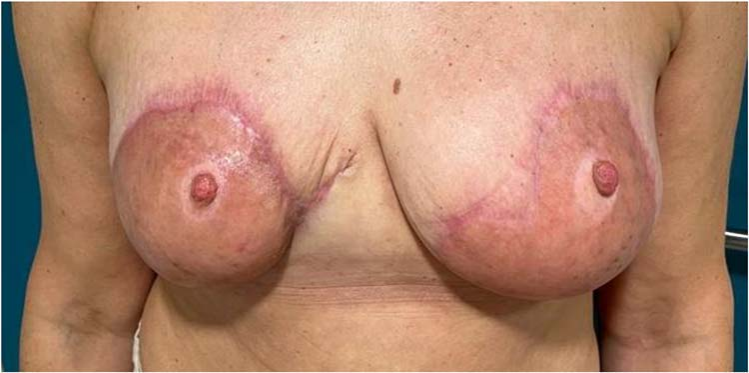
Figure representing the aesthetic outcome several weeks after multiple debridement Integra and split thickness skin graft application

The aesthetic outcome (Fig. 7) was satisfactory to the patient. The reconstructed skin was soft and pliable, and the patient had full sensation of her nipples. The patient was offered a symmetrizing procedure but declined.

## DISCUSSION

Necrotizing fasciitis is an infective process that ultimately results in necrosis of the fascia as well as subcutaneous tissue, while sparing the muscular layer [1–3]. This is a rapidly progressive process, spreading through facial planes and being potentially fatal [4]. Bacterial toxins are produced which lead to widespread necrosis of tissue adjacent to the fascial planes and can therefore lead to systemic toxicity and sepsis [2, 5].

Necrotizing fasciitis risks include:

- Diabetes mellitus
- An immunocompromised state
- Peripheral vascular disease
- Underlying malignancy
- Chronic renal failure
- Intravenous drug use [5, 6].

Necrotizing fasciitis of the breast can be idiopathic [7] or secondary to other causes including elective mastectomy (total or partial) [8, 9], following a needle core biopsy [10], penetrating injury [11], human bites [10], anticoagulation therapy [12], in patients with puerperal sepsis [13], topical belladonna application [14] and breast tumour with mammary infarcts in pregnant patients [15].

Necrotizing fasciitis can be classified into four main categories based on etiology:

- Type I: polymicrobial in nature [16]—potential causes include a wide variety of bacterial agents including Gram-positive cocci such as *Staphylococcus* and *Streptococcus* species, Gram-negative bacilli such as *Klebsiella* species and *Escherichia coli* and anaerobes [17, 18]. This type commonly occurs in the perineum and abdomen with the etiological bacterial agents introduced through either blunt or penetrating trauma or surgical incisions [5, 16].
- Type II: monomicrobial in nature, being caused by group A, β-hemolytic streptococcal species such as *Streptococcus pyogenes* [5]. This can also be associated with concurrent staphylococcal infection [6]. In contrast to type I, type II is associated with minor injuries or breaks in the skin. Furthermore, this type most commonly affects immunocompetent hosts [18].
- Type III: caused by monomicrobial infection with Gram-negative bacteria, such as *Clostridium*, *Klebsiella* or *Vibrio* species [16]. The latter species are commonly transmitted to humans by fish or other marine hosts [16]. This is uncommon.
- Type IV: caused by fungal infections and is common following burns or large traumatic wounds [19].

The diagnosis of necrotizing fasciitis can be challenging and a high index of suspicion is required to initiate prompt surgical management and decrease morbidity and mortality [19]. Swelling, erythema, pain out of proportion to clinical signs and development of fluid filled bullae are common signs that should be looked out for [16, 19]. A common differential diagnosis is cellulitis. The key pathological findings in necrotizing fasciitis are: full thickness skin necrosis; perivasculitis and vasculitis, often with accompanying fibrinoid necrosis and thrombus formation; and the presence of bacterial or fungal elements on ancillary stains [16].

In the breast, necrotizing fasciitis is most commonly unilateral [1] and may pose a diagnostic challenge. The variable thickness of the underlying breast tissue between the skin and the fascia results in a delayed cutaneous reaction and therefore widespread infection prior to diagnosis and surgical management [20]. Moreover, it can be misdiagnosed as inflammatory breast cancer, cellulitis, mastitis or breast abscess [21].

The dense blood supply network that supplies the breast ultimately acts as a protective factor. This includes branches from the thoracoacromial branches, lateral thoracic, lateral mammary branches, internal thoracic and internal mammary arteries [22]. This supply delays cutaneous involvement and hence also delaying presentation due to the angiothrombotic element of the necrotizing fasciitis [23]. Normally, the majority of patients present weeks after the initial commencement of symptoms due to the mentioned reasons. In the case discussed in this report, the patient had been operated on for a bilateral breast reduction 10 days previously. The initial diagnosis was that of an infected hematoma, but when patchy skin necrosis was observed on both breasts and the patient showed signs of sepsis, a diagnosis of necrotizing fasciitis was postulated.

Surgical findings include extensive necrosis along fascial planes [21]. During wound exploration debridement, tissue must be assessed for viability and all areas of necrosis must be excised meticulously [18]. Although deep wounds were approximated in this scenario, necrotic superficial layers were left open to allow for repeated examination and/or debridement. In this case, the patient required repeated debridement before covering the wound with Integra and a split thickness skin graft. Most cases require repeated debridement and sometimes even mastectomy. An interesting fact of this case was that the nipple was completely spared, and the patient still had sensation.

In some instances, patients have undergone US, MRI or computed tomography scans to determine the extent of necrotic tissue and ultimately the cause of the necrotizing fasciitis. Since in this case the patient presented with signs of systemic toxicity, urgent debridement was required, and timely investigations were left out.

A thorough literature review suggests that staged debridement as opposed to immediate mastectomy as a treatment strategy is a better approach. Although breast conservation is possible in most cases such as this one, early radical mastectomy can be life-saving in severe cases of necrotizing fasciitis [21, 24, 25]. If the defect is large and not amenable to primary closure, a vacuum-assisted closure dressing can assist in wound closure [11] but may often require a split-thickness skin graft to cover the defect or other advanced reconstructive procedures.

Necrotizing fasciitis of the breast is rarely diagnosed with relatively few cases described in the literature. It was first described in the literature by Shah *et al*. in 2001 [26], and only a handful of cases have been published since. Of these cases, only six reports of primary necrotizing fasciitis of the breast occurring in non-lactating, previously healthy women. These cases are described in Table 1.

**Table 1:** Existing case reports of primary necrotizing fasciitis in non-lactating, previously healthy women

**Author (year)**	**Patient age**	**Surgical treatment used**
Rajakannu *et al*. (2006) [21]	50	Mastectomy
Wong *et al*. (2008) [20]	38	Quadrantectomy
Keune *et al*. (2008) [24]	47	Mastectomy
Soliman *et al*. (2011) [6]	61	Debridement
Yang *et al*. (2015) [27]	30	Debridement
Marongiu *et al*. (2016) [4]	39	Debridement + hyperbaric oxygen

Moreover, no cases were found of necrotizing fasciitis post bilateral breast reduction. This is a unique case as it is the first European case that would be published. In addition, this case highlights the need for pre- and post-operative patient education to focus on the importance of recognizing important signs of infection and deterioration. Early diagnosis can assist in circumventing the significant morbidity and mortality associated with necrotizing fasciitis. Moreover, the successful treatment of this patient highlights the importance of conservative surgical management as opposed to full mastectomy.

## CONCLUSION

This case report represents the first necrotizing fasciitis post bilateral breast reduction. Breast necrotizing fasciitis is rare. Its exact etiology is variable and multifactorial. Delayed presentation is common and unfortunately very detrimental. Diagnosis of this infective process is ultimately on the basis of clinical but can also be aided with laboratory findings, imaging, and culture findings. The gold standard in the management of necrotizing fasciitis is prompt surgical debridement along with broad-spectrum antibiotic treatment.

## Data Availability

The data that support the findings of this case report are available in the hospital department mentioned above. The authors confirm that the data supporting the findings of this case report are available within the article and its supplementary materials.
